# Prevalence and molecular characterization of *Cryptosporidium* spp. in dairy cattle in Central Inner Mongolia, Northern China

**DOI:** 10.1186/s12917-023-03696-z

**Published:** 2023-08-25

**Authors:** Li Zhao, Hai-Liang Chai, Ming-Yuan Wang, Zhan-Sheng Zhang, Wen-Xiong Han, Bo Yang, Yan Wang, Shan Zhang, Wei-Hong Zhao, Yi-Min Ma, Yong-Jie Zhan, Li-Feng Wang, Yu-Lin Ding, Jin-Ling Wang, Yong-Hong Liu

**Affiliations:** 1 College of Veterinary Medicine, Inner Mongolia Agricultural University, Hohhot, China; 2 Key Laboratory of Clinical Diagnosis and Treatment Technology in Animal Disease, Ministry of Agriculture and Rural Affairs, Hohhot, China; 3 Inner Mongolia Saikexing Reproductive Biotechnology (Group) Co.,Ltd, Hohhot, China; 4 Animal Disease Control Center of Ordos, Ordos, China

**Keywords:** *Cryptosporidium*, Prevalence, Molecular characterization, China

## Abstract

**Background:**

*Cryptosporidium* is a gastrointestinal protozoan that widely exists in nature, it is an established zoonotic pathogen. Infected cattle are considered to be associated with cryptosporidiosis outbreaks in humans. In the present study, we aimed to assess the prevalence and species distribution of *Cryptosporidium* in dairy cattle in Central Inner Mongolia.

**Methods:**

We focused on the small subunit ribosomal RNA gene (*SSU rRNA*) of *Cryptosporidium* and 60-kDa glycoprotein gene (*gp60*) of *Cryptosporidium parvum*. We collected 505 dairy cattle manure samples from 6 sampling sites in Inner Mongolia in 2021; the samples were divided into 4 groups based on age. DNA extraction, polymerase chain reaction (PCR), sequence analysis, and restriction fragment length polymorphism (RFLP) using SspI and MboII restriction endonucleases were performed. RFLP analysis was performed to determine the prevalence and species distribution of *Cryptosporidium*.

**Results:**

*SSU rRNA* PCR revealed that the overall prevalence of *Cryptosporidium* infection was 29.90% (151/505), with a prevalence of 37.67% (55/146) and 26.74% (96/359) in diarrheal and nondiarrheal samples, respectively; these differences were significant. The overall prevalence of *Cryptosporidium* infection at the 6 sampling sites ranged from 0 to 47.06% and that among the 4 age groups ranged from 18.50 to 43.81%. *SSU rRNA* sequence analysis and RFLP analysis revealed the presence of 4 *Cryptosporidium* species, namely, *C. bovis* (44.37%), *C. andersoni* (35.10%), *C. ryanae* (21.85%), and *C. parvum* (11.92%), along with a mixed infection involving two or three *Cryptosporidium* species. *Cryptosporidium bovis* or *C. andersoni* was the most common cause of infection in the four age groups. The subtype of *C. parvum* was successfully identified as IIdA via *gp60* analysis; all isolates were identified as the subtype IIdA19G1.

**Conclusions:**

To the best of our knowledge, this is the first report of dairy cattle infected with four *Cryptosporidium* species in Inner Mongolia, China, along with a mixed infection involving two or three *Cryptosporidium* species, with *C. bovis* and *C. andersoni* as the dominant species. Moreover, this is the first study to identify *C. parvum* subtype IIdA19G1 in cattle in Inner Mongolia. Our study findings provide detailed information on molecular epidemiological investigation of bovine cryptosporidiosis in Inner Mongolia, suggesting that dairy cattle in this region are at risk of transmitting cryptosporidiosis to humans.

## Background

*Cryptosporidium* is an important protozoan pathogen [1–5] that infects humans and animals (domestic animals, mammals, marsupials, rabbits, rodents, fish, birds, reptiles, and amphibians) [6, 7]; it is the fifth most important foodborne parasite globally [8]. Cryptosporidiosis is a global parasitic disease [6, 9, 10] and presents as a large-scale foodborne and waterborne outbreak [1, 4, 6, 11–13]. *Cryptosporidium* causes symptoms such as self-limiting diarrhea in humans [1, 6, 14] and is the second most important diarrhea-causing pathogen in children after rotavirus [4, 13, 15]; moreover, it may be lethal to immunosuppressed individuals [6]. In farm animals, cryptosporidiosis is the main cause of diarrhea in neonatal livestock and remains one of the most important diseases affecting neonatal calves [4]. This disease leads to reduced weight gain, poor feed conversion [2], and substantial production losses [4, 9, 10] in juvenile animals as well as significant mortality in preweaned calves [9, 16]. Adult livestock typically exhibit less severe and asymptomatic infections; however, they are epidemiologically important as cryptic carriers of parasites as they may lead to herd level reinfections [4]. Infected cattle, particularly preweaned calves [10, 13], are potential important reservoirs for environmental contamination and human infections [2, 4]. Moreover, only a few drugs with poor therapeutic efficacy are available for cryptosporidiosis, and no vaccines have yet been developed [1, 6, 9, 17].

To date, at least 44 valid *Cryptosporidium* species and approximately 120 genotypes have been reported globally [1, 6, 9]; of these, 29 are mammalian species, with at least 19 species and 4 genotypes reported in humans. *Cryptosporidium hominis* and *C. parvum* are the most abundant and important species involved in human infections [1, 9, 12]. In cattle, at least 12 *Cryptosporidium* species have been reported globally [2], with *C. parvum*, *C. bovis*, *C. ryanae*, and *C. andersoni* being the dominant species [2, 4, 12, 18–20]. In preweaned calves, *C*. *parvum* is the most dominant species [19], which occurs almost exclusively. In China, at least 10 *Cryptosporidium* species have been identified in cattle, with the abovementioned 4 species being the most common [2, 13]. However, *C. bovis* is the dominant species in preweaned calves [2, 13, 16, 21], and *C. andersoni* is the dominant species in postweaned, juvenile, and adult cattle [2]; *C. parvum* is mainly found in preweaned calves [2, 5]. Moreover, *C. ryanae* is identified in preweaned calves [22–25], whereas *C. bovis* and *C. ryanae* are common in postweaned calves [23]. In dairy cattle, *C. andersoni* is the most common species [2]. Zoonotic cryptosporidiosis is mainly caused by *C.parvum* [12, 16, 20, 26], which is found in various animals (ruminants, equine animals, rodents, and primates) [12]. Over 20 subtype families of 60-kDa glycoprotein gene (*gp60*) in *C. parvum* have been identified [1, 16, 18], of which IIa, IIc, and IId are the most widely recognized subtype families. Subtype IIc appears to be anthroponotically transmitted, whereas subtypes IIa and IId are zoonotically transmitted [1, 5, 12, 27–31]. In most countries including industrialized countries, cattle are mainly infected with the subtype IIa [32, 33]; however, *C. parvum* infections in cattle in China are exclusively caused by the subtype IId, of which IIdA15G1 and IIdA19G1 are the most common subtypes [5, 16, 18, 20].

The global prevalence of *Cryptosporidium* infection is 7.6% in humans, with an average prevalence of 4.3% and 10.4% in developed and developing countries, respectively [34]. In China, the average prevalence of *Cryptosporidium* infection in humans was 2.97% in 27 provinces between 1987, when it was first reported, and 2018 [17]. Between 1984 and 2016, 18.9% of common livestock (cattle, goats, sheep, horses, pigs, and buffaloes) were infected with *Cryptosporidium* spp. globally; moreover, domestic hoofed animals (camels, yaks, donkeys, alpacas, and llamas) exhibited a *Cryptosporidium* infection prevalence of 13.6%. Conventional microscopy (CM) and polymerase chain reaction (PCR) revealed that 23.4% of common livestock were positive for *Cryptosporidium* spp. infection. The pooled prevalence of *Cryptosporidium* infection in cattle was 22.5% (CM) or 29.1% (PCR). The prevalence of *Cryptosporidium* infection in livestock in different regions is mostly in the range of 5–30%. The highest and lowest prevalence of *Cryptosporidium* infection have been reported in America (26%) and Africa (14%), respectively; its highest prevalence observed in New Zealand is lower than that in other regions. Among 53 countries, livestock in Canada (60%) exhibited the highest infection rate, whereas those in China, Thailand, and Germany (8%) had the lowest infection rates [4]. In 1986, the first report of bovine *Cryptosporidium* infection in China was published in Lanzhou, Gansu Province [35]. Until 2016, *Cryptosporidium* species were distributed in 19 provinces in China, with an overall infection rate of 11.9% and average infection rate of 10.44% in dairy cattle [2]. During the same period, the overall infection rate of bovine *Cryptosporidium* in China was 14.50% and the prevalence in dairy cattle was 13.98% [13]. The pooled prevalence of *Cryptosporidium* infection in dairy cattle in 23 provinces in China was 17.0% during 2008–2018; this prevalence of varied among different provinces in China, with the highest and lowest prevalence observed in Heilongjiang (35.6%) and Tianjin (4.3%), respectively [9]. Inner Mongolia is located on the northern border of China, spanning 28°52′ longitude from east to west, with a linear distance of > 2400 km, and 15°59′ latitude from north to south, with a linear distance of 1700 km. Currently, only two studies in Chinese in Inner Mongolia have reported the prevalence of *Cryptosporidium* infection in dairy cattle to be 24.56% (14/57) [36] and 14.92% (44/295) [37] using CM and PCR, respectively, and only *C. andersoni* was identified in the latter. In the present study, we aimed to investigate the prevalence and species distribution of *Cryptosporidium* in dairy cattle in Central Inner Mongolia.

## Methods

### Study areas and sample collection

From March to September 2021, 505 fresh fecal samples were randomly collected from 4 intensive dairy farms and 2 free-ranging dairy farms in the vicinity of Tumed Left Banner, Horinger County, Togtoh County, Dalad Banner, and Hanggin Rear Banner (113°34′E–118°28′E, 24°29′N–30°04′N) in Central Inner Mongolia. The fecal samples were collected via rectal sampling from dairy cattle or from the inner top layer of the fresh feces. These samples were obtained from 103 preweaned calves (aged 0–60 days), 105 postweaned calves (aged 61–180 days), 124 young cattle (aged 181–360 days), and 173 adult cattle (aged > 361 days). Information regarding whether the animals experienced diseases such as diarrhea was recorded during sampling; the samples were transferred to the laboratory and stored at 4 °C until later use.

### DNA extraction and PCR amplification

DNA was extracted from 505 fecal samples in a biosafety cabinet using E.Z.N.A® Stool DNA Kit (Omega Biotek, Norcross, GA, USA) according to the manufacturer’s instructions and was stored at − 20 °C for subsequent experiments.

The extracted DNA was used as a template and the small subunit ribosomal RNA gene (*SSU rRNA*) of *Cryptosporidium* [38] was amplified via nested PCR (annealing temperatures of 55 and 58 °C) using Premix Taq™ (TaKaRa Taq™ Version 2.0 plus dye) (TaKaRa, Beijing, China). Positive PCR products were sent to a commercial company (Sangon Biotech, Shanghai, China) for sequencing. Simultaneously, *SSU rRNA* positive amplification products were subjected to restriction fragment length polymorphism (RFLP) analysis using the restriction enzymes (SspI and MboII (TaKaRa) [39]. The results of RFLP and *SSU rRNA* gene bidirectional sequencing analyses were used to analyze the extracted DNA of *C. parvum* and perform nested PCR (annealing temperatures of 52 °C and 50 °C) of *gp60* [38]. The sequencing results of *gp60* were used to identify the subtype of *C. parvum* [40].

### Sequence analysis

The sequences were aligned with reference sequences downloaded from GenBank (http://www.ncbi.nlm.nih.gov) using the MEGA 5.0 software (http://www.megasoftware.net/). The BLAST online platform was used to analyze the sequencing results. Phylogenetic analyses were performed using the concatenated dataset of *gp60* sequences. Using the NeighborJoining (NJ) algorithm, phylogenetic trees were constructed based on a matrix of evolutionary distances calculated via the Kimura 2-parameter model of the MEGA 7.0 software. Bootstrap analysis was performed using 1000 replicates to assess the robustness of clusters.

### Statistical analysis

Chi-square test was performed and 95% confidence interval (CI) was determined using SPSS Statistics 21.0 (IBM Corp., New York, NY, USA) to compare *Cryptosporidium* infection rates among different sampling sites and age groups as well as between the diarrheal and nondiarrheal groups. A two-tailed *p-value* of < 0.05 was considered to indicate statistical significance.

## Results

### *Cryptosporidium* infection status

For the *SSU rRNA of Cryptosporidium*, the PCR amplification of 505 samples yielded positive results in 151 samples, with the overall prevalence of *Cryptosporidium* infection being 29.90% (151/505). The overall prevalence in diarrheal and nondiarrheal samples was 37.67% (55/146) and 26.74% (96/359), respectively (Table 1); this difference was significant, with an odds ratio (OR) of 1.656 (95% CI: 1.101–2.491, p = 0.015).

The overall prevalence of *Cryptosporidium* infection in all samples at the 6 sampling sites was 39.29% (54/140), 24.55% (27/110), 22.50% (27/120), 31.82% (35/100), 47.06% (8/17), and 0% (0/8). A significant difference was observed between Tumed Left Banner 1 and Tumed Left Banner 2, with an OR of 1.930 (95% CI: 1.112–3.351, p = 0.019). Moreover, there was a highly significant difference between Tumed Left Banner 1 and Horinger County, with an OR of 2.136 (95% CI: 1.251–3.738, p = 0.005). Further, no significant differences were observed between the other two farms (p > 0.05). The prevalence of *Cryptosporidium* infection in diarrheal samples at the 6 sampling sites was 45.45% (25/55), 50% (6/12), 27.50% (11/40), 33.33% (13/39), 0% (0/0), and 0% (0/0; Table 1); only Tumed Left Banner 2 farm showed significant difference in prevalence between diarrheal and nondiarrheal samples, with an OR of 3.667 (95% CI: 1.072–12.547, p = 0.030; Table 1).

The overall prevalence of *Cryptosporidium* infection in all samples was 27.18% (27/103), 43.81% (46/105), 37.10% (46/124), and 18.50% (32/173) in preweaned calves, postweaned calves, young cattle, and adult cattle, respectively. A highly significant difference was observed in the prevalence between pre- and postweaned calves [OR of 0.456 (95% CI: 0.254–0.817, p = 0.008)], between postweaned calves and adult cattle [OR of 3.435 (95% CI: 1.994–5.919, p = 0.000)], and between young and adult cattle [OR of 2.599 (95% CI: 1.531–4.411, p = 0.000)]. The differences in prevalence between the remaining two age groups were not significant (p > 0.05). The prevalence of *Cryptosporidium* infection in diarrheal samples was 30% (9/30), 45.59% (31/68), 50% (10/20), and 17.86% (5/28) in preweaned calves, postweaned calves, young cattle, and adult cattle, respectively, with no significant difference being observed between prevalence in diarrheal and nondiarrheal samples within each age group (Table 1).

### RFLP and sequence analysis

Overall, 151 PCR amplification products of *SSU rRNA* gene were analyzed via RFLP, and the results were combined with those of sequencing analysis, four *Cryptosporidium* species were identified, namely, *C. bovis* (44.37%, 67/151), *C. andersoni* (35.10%, 53/151), *C. ryanae* (21.85%, 33/151), and *C. parvum* (11.92%, 18/151) along with the presence of mixed infections involving two or three *Cryptosporidium* species (Table 1). Three intensive dairy farms were infected with four *Cryptosporidium* species, one intensive dairy farm was infected with three *Cryptosporidium* species, and one free-ranging dairy farm was infected with two *Cryptosporidium* species.

Preweaned calves were frequently infected with *C. b*ovis (15/27), followed by *C. parvum* (14/27), whereas postweaned calves were often infected with *C. bovis* (29/46), followed by *C. ryanae* (14/27). Young cattle were mostly infected with *C. andersoni* (28/46), followed by *C. bovis* (14/46), whereas adult cattle were often infected with *C. andersoni* (21/32), followed by *C. bovis* (9/32), but not with *C. parvum*. Infection with *C. parvum* alone occurred only in preweaned calves, whereas infections with the other three *Cryptosporidium* spp. alone were observed in all four age groups. Mixed infections and four *Cryptosporidium* species were identified in all age groups except adult cattle (Table 1).

**Table 1:** Prevalence of *Cryptosporidium* infection and information regarding *Cryptosporidium* species

Farm	Samples size	Age				Total	*p*-value	OR (95% CI)	*Cryptosporidium* species (No.)	*C. parvum* gp60 subtype (No.)
		preweaned calves	postweaned calves	young cattle	adult cattle					
**Tumd Left Banner 1**	Samples size (Diarrheal samples size )	20 (8)	40 (21)	40 (9)	40 (17)	140 (55)	0.178	1.609 (0.803–3.224)	*C. parvum* (1); *C. bovis* (26); *C. ryanae* (10); *C. andersoni* (11); *C. parvum* + *C. ryanae* (1)^a^; *C. bovis* + *C. ryanae* (4)^a^; *C. parvum* + *C. ryanae* + *C. andersoni* (1)^a^	IIdA19G1 (2)
	Positive samples size (Diarrheal positive samples size )	10 (5)	23 (13)	15 (5)	6 (2)	54 (25)				
	Overall prevalence (Prevalence of diarrheal samples) (%)	50% (62.5%)	57.5% (61.90%)	37.5% (55.56%)	15% (11.76%)	38.57% (45.45%)				
**Tumd Left Banner 2**	Samples size (Diarrheal samples size )	30 (2)	20 (9)	20 (1)	40 (0)	110 (12)	0.030	3.667 (1.072–12.547)	*C. parvum* (3); *C. bovis* (12); *C. ryanae* (5); *C. andersoni* (5); *C. bovis* + *C. ryanae* (1)^a^; *C. parvum* + *C. ryanae* + *C. andersoni* (1)^a^	IIdA19G1 (4)
	Positive samples size (Diarrheal positive samples size )	8 (0)	9 (5)	7 (1)	3 (0)	27 (6)				
	Overall prevalence (Prevalence of diarrheal samples) (%)	26.67% (0)	45% (55.56%)	35% (100%)	7.5% (0)	24.55% (50%)				
**Horinger County**	Samples size (Diarrheal samples size )	23 (8)	20 (18)	41 (7)	36 (7)	120 (40)	0.354	1.517 (0.627–3.673)	*C. parvum* (4); *C. bovis* (4); *C. ryanae* (2); *C. andersoni* (9); *C. parvum* + *C. bovis* (3)^a^; *C. parvum* + *C. ryanae* (2)^a^; *C. bovis* + *C. ryanae* (1)^a^; *C. parvum* + *C. bovis* + *C. ryanae* (2)^a^	IIdA19G1 (9)
	Positive samples size (Diarrheal positive samples size )	9 (4)	4 (4)	11 (3)	3 (0)	27 (11)				
	Overall prevalence (Prevalence of diarrheal samples) (%)	39.13% (50%)	20% (22.22%)	26.83% (42.86%)	8.33% (0)	22.5% (27.5%)				
**Togtoh County**	Samples size (Diarrheal samples size )	30 (12)	20 (20)	20 (3)	40 (4)	110 (39)	0.861	0.928 (0.400–2.153)	*C. bovis* (11); *C. ryanae* (3); *C. andersoni* (21)	-
	Positive samples size (Diarrheal positive samples size )	0 (0)	9 (9)	11 (1)	15 (3)	35 (13)				
	Overall prevalence (Prevalence of diarrheal samples) (%)	0% (0)	45% (45%)	55% (33.33%)	37.5% (75%)	31.82% (33.33%)				
**Dalad Banner**	Samples size (Diarrheal samples size )	0 (0)	5 (0)	3 (0)	9 (0)	17 (0)	-	-	*C. bovis* (3); *C. andersoni* (5)	-
	Positive samples size (Diarrheal positive samples size )	0	1 (0)	2 (0)	5 (0)	8 (0)				
	Overall prevalence (Prevalence of diarrheal samples) (%)	0.00% (0)	20% (0)	66.67% (0)	55.56% (0)	47.06% (0)				
**Hanggin Rear Banner**	Samples size (Diarrheal samples size )	0 (0)	0 (0)	0 (0)	8 (0)	8 (0)	-	-	-	-
	Positive samples size (Diarrheal positive samples size )	0 (0)	0 (0)	0 (0)	0 (0)	0 (0)				
	Overall prevalence (Prevalence of diarrheal samples) (%)	0.00% (0)	0.00% (0)	0.00% (0)	0% (0)	0% (0)				
**Total**	Samples size (Diarrheal samples size )	103 (30)	105 (68)	124 (20)	173 (28)	505 (146)	-	-	-	-
	Positive samples size (Diarrheal positive samples size )	27 (9)	46 (31)	46 (10)	32 (5)	151 (55)				
	Overall prevalence (Prevalence of diarrheal samples) (%)	27.18% (30%)	43.81% (45.59%)	37.10% (50%)	18.50% (17.86%)	29.90% (37.67%)				
***p*** **-value**	-	0.575	0.618	0.192	0.924	-	-	-	-	-
**OR (95% CI)**	-	1.310(0.509–3.369)	1.229(0.546–2.766)	1.889(0.720–4.959)	0.950(0.331–2.725)					
***Cryptosporidium*** **species (No.)**	-	*C. parvum* (8); *C. bovis* (10); *C. ryanae* (2); *C. andersoni* (1); *C. parvum* + *C. bovis* (3)^a^; *C. parvum* + *C. bovis* + *C. ryanae* (2)^a^; *C. parvum* + *C. ryanae* + *C. andersoni* (1)^a^	*C. bovis* (26); *C. ryanae* (14); *C. andersoni* (2); *C. parvum* + *C. ryanae* (1)^a^; *C. bovis* + *C. ryanae* (3)^a^	*C. bovis* (11); *C. ryanae* (2); *C. andersoni* (27); *C. parvum* + *C. ryanae* (2)^a^; *C. bovis* + *C. ryanae* (3)^a^; *C. parvum* + *C. ryanae* + *C. andersoni* (1)^a^	*C. bovis* (9); *C. ryanae* (2); *C. andersoni* (21)	-	-	-	*C. parvum* (8); *C. bovis* (56); *C. ryanae* (20); *C. andersoni* (51); *C. parvum* + *C. bovis* (3)^a^; *C. parvum* + *C. ryanae* (3)^a^; *C. bovis* + *C. ryanae* (6)^a^; *C. parvum* + *C. bovis* + *C. ryanae* (2)^a^; *C. parvum* + *C. ryanae* + *C. andersoni* (2)^a^	-
***C. parvum*** **gp60 subtype (No.)**	-	IIdA19G1 (13)	-	IIdA19G1 (2)	-	-	-	-	-	IIdA19G1 (15)

dash (–) indicates that no data were obtained.

^a^ indicates Mixed infections.

The abovementioned four *Cryptosporidium* spp. were identified in both diarrheal and nondiarrheal samples; *C. bovis* (33/55) was the most frequently detected species in diarrheal samples, followed by *C. ryanae* (16/55), whereas *C. andersoni* (43/96) was the most frequently detected species in nondiarrheal samples, followed by *C. bovis* (34/96) (Table 2).

**Table 2:** *Cryptosporidium* infection in different clinical samples of dairy cattle

Clinical symptoms	Samples size	No. positive for *Cryptosporidium* (%)	*Cryptosporidium* species (No.)
Diarrheal	146	55(37.67%)	*C. parvum* (2); *C. bovis* (26); *C. ryanae* (8); *C. andersoni* (10); *C. parvum* + *C. bovis* (1)^a^; *C. parvum* + *C. ryanae* (2)^a^; *C. bovis* + *C. ryanae* (5)^a^; *C. parvum* + *C. bovis* + *C. ryanae* (1)^a^
Nondiarrheal	359	96 (26.74%)	*C. parvum* (6); *C. bovis* (30); *C. ryanae* (12); *C. andersoni* (41); *C. parvum* + *C. bovis* (2)^a^; *C. parvum* + *C. ryanae* (1)^a^; *C. bovis* + *C. ryanae* (1)^a^; *C. parvum* + *C. bovis* + *C. ryanae* (1)^a^; *C. parvum* + *C. ryanae* + *C. andersoni* (2)^a^
**Total**	505	151 (29.90%)	*C. parvum* (8); *C. bovis* (56); *C. ryanae* (20); *C. andersoni* (51); *C. parvum* + *C. bovis* (3)^a^; *C. parvum* + *C. ryanae* (3)^a^; *C. bovis* + *C. ryanae* (6)^a^; *C. parvum* + *C. bovis* + *C. ryanae* (2)^a^; *C. parvum* + *C. ryanae* + *C. andersoni* (2)^a^

^a^ indicates Mixed infections.

**Identification of*C. parvum*subtype**.

**Fig. 1:**
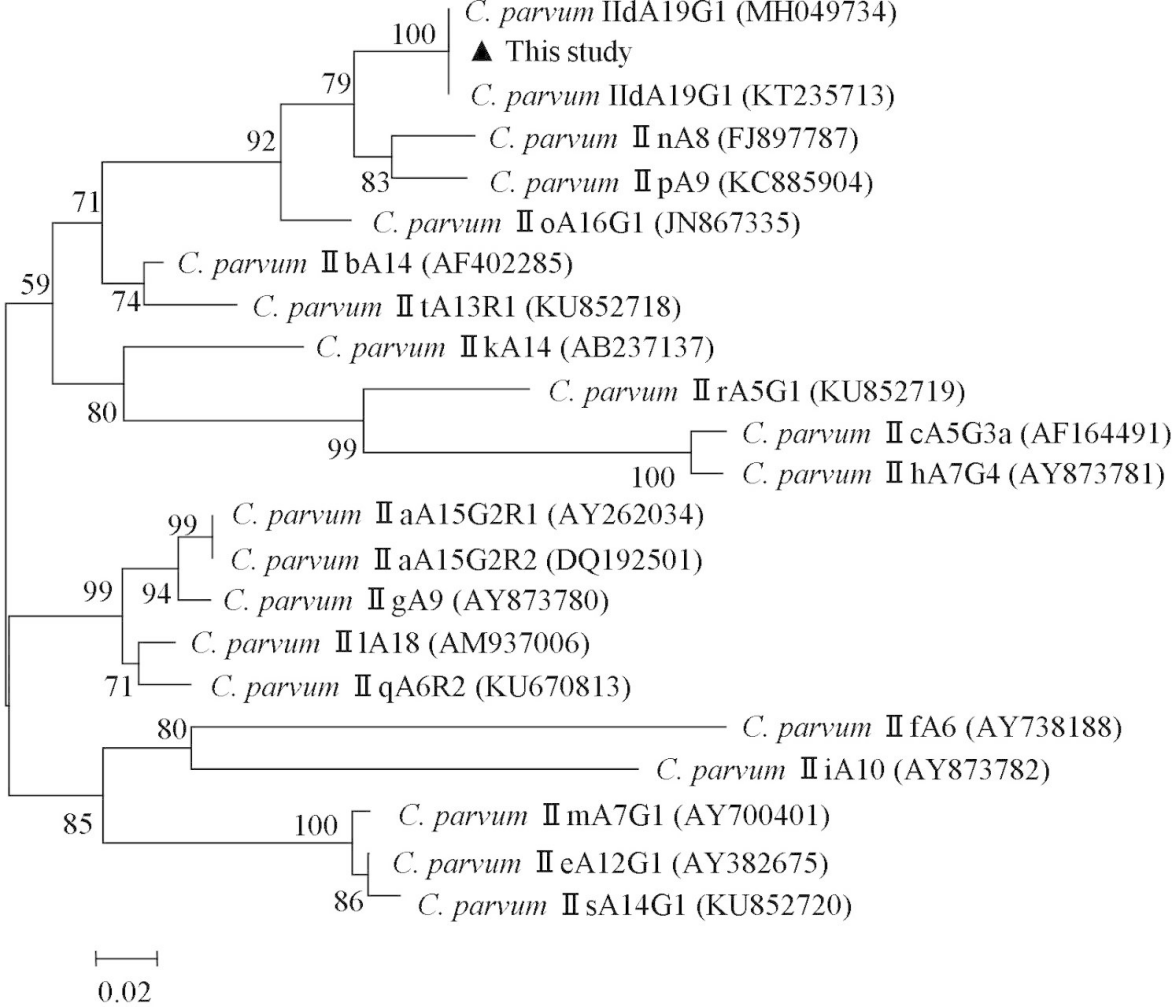
A phylogenetic tree of *Cryptosporidium parvum* based on gp60 sequences. The phylogenetic tree was constructed via a NeighborJoining analysis of genetic distances calculated using the Kimura 2-parameter model. Percent bootstrap values of > 50% from 1000 replicates are shown to the left of nodes. The isolates indicated as black triangles (▲) represent the subtype IId, which was identified in cattle in this study

In total, 15 *gp60* sequences were analyzed in this study; phylogenetic analysis of *gp60* sequences based on *C. parvum* showed that *gp60* obtained in the present study belonged to the same branch as the reference subtype IId (Fig. 1) and were successfully identified as *C. parvum* subtype family IIdA19G1 (Table 1).

## Discussion

To date, several studies worldwide have reported *Cryptosporidium* infection in cattle [4]. Bovine cryptosporidiosis is also widespread in China [2, 10, 13]. In the current study, a molecular epidemiological systematic investigation of *Cryptosporidium* was conducted using 505 dairy cattle feces samples obtained from 6 sampling sites in Central Inner Mongolia, thereby providing information on *Cryptosporidium* infection in cattle in Inner Mongolia. Furthermore, our study also reconfirmed the occurrence of cryptosporidiosis in animals in Inner Mongolia [41–47]. The overall prevalence of *Cryptosporidium* infection in dairy cattle was found to be 29.90% (151/505), which was close to the global pooled prevalence of 29.1% for bovine cryptosporidiosis [4] but higher than that in dairy cattle in China (10.44% [2], 13.98% [12], or 17.0% [9]). The prevalence observed in the present study was similar to that in Northeastern China (29.8%) and higher than that in Central China (16. 9%), Eastern China (17.4%), Northern China (15.7%), Northwestern China (15.8%), Southern China (9.5%), and Southwestern China (13.7%) [10]. Based on the single reports of the prevalence of *Cryptosporidium* infection in dairy cattle in various provinces of China, the prevalence observed in the current study was lower than that in Shanghai (37.0%) [25], Xinjiang (38.4% [48] and 52.0% [22]), Heilongjiang (47.68%) [49], Taiwan (37.6%) [50], and Henan (33.89%) [51] but higher than that in other regions of China [3, 52–69]; moreover, it was higher than the prevalence reported in only two surveys on *Cryptosporidium* in Inner Mongolia [36, 37].

In the current study, the overall prevalence of *Cryptosporidium* infection was between 22.50% and 47.06% at the five sampling sites. The difference in prevalence between Tumed Left Banner 1 and Tumed Left Banner 2 was significant, and that between Tumed Left Banner 1 and Horinger County was highly significant. The maximum prevalence in other provinces in China also differed significantly, from 2.6% (Hebei/Tianjin) [65] to 100% (Heilongjiang) [49]. However, it is difficult to compare the prevalence data as they are influenced by various factors, including geographic conditions, climate, sanitation conditions, rearing conditions, total number of samples, sampling season, age of animals, and diagnostic methods [2, 4]. In addition, early infection in an area may be attributed to poor breeding conditions and backward management. Reportedly, the subsequent gradual decline in the prevalence of *Cryptosporidium* infection is owing to the implementation of effective control regulations [10]. However, the increase in the prevalence of *Cryptosporidium* infection may be attributed to high stocking densities due to concentrated animal feeding operations (CAFOs) [4, 16].

In the present study, both the overall *Cryptosporidium* infection prevalence and prevalence in Tumed Left Banner 2 field were correlated with whether the sampled animals had diarrhea. This is consistent with the combined Chinese study reporting a higher prevalence of *Cryptosporidium* infection in cattle with diarrhea than in those without diarrhea [10, 13]. However, in the current study, the difference in prevalence between diarrheal and nondiarrheal samples within each age group was not significant. It is well known that diarrhea is a common clinical symptom of multiple diseases, and its causative agents include bacteria, viruses, parasites or other possible factors [70, 71]. Therefore, it is difficult to compare the prevalence of a single pathogen in diarrheal and nondiarrheal samples.

In the present study, among the four age groups, the overall prevalence of *Cryptosporidium* infection was the highest in postweaned calves (43.81%), followed by young cattle (37.10%), preweaned calves (27.18%), and adult cattle (18.50%), with significant differences. Age-specific prevalence observed in this study was significantly higher than the pooled prevalence reported in China, according to the combined pre-2016 data on preweaned calves (19.5%), juveniles (10.69%), postweaned juveniles (9.0%), and adult cattle (4.94%) [2]; this did not reflect the decrease in infection rate with an increase in age of animals, as reported in the literature [13]. In China, some studies have also reported a high prevalence of *Cryptosporidium* infection in postweaned calves [58, 64, 68]; however, there are several reports showing high prevalence in preweaned calves [50–52, 54, 55, 60, 63, 65]. Indeed, some studies have reported inconsistencies in the time interval between preweaned and postweaned cows or there was a lack of accurate information regarding the age of sampled cows. If calves aged < 3 months are classified as preweaned calves, it was observed that some postweaned calves should have been classified as preweaned calves. As mentioned above, several factors affect *Cryptosporidium* infection, including prevalence, age distribution, and the presence or absence of diarrhea. In addition, it is related to the nonspecific immunity acquired through factors such as breast milk, immature immune defenses [10], different feeding patterns [4], and oocyte activity [2, 4, 5, 20].

To the best of our knowledge, this is the first study to simultaneously identify and report four *Cryptosporidium* species in cattle in Inner Mongolia; the species identified are consistent with the dominant *Cryptosporidium* species reported in cattle worldwide [2, 4, 9, 12, 18–20] and in China [2, 3, 12, 13, 22, 49, 52, 55, 57, 60, 61, 63]. Moreover, this study is the first to detect mixed infections involving two or three *Cryptosporidium* species in dairy cattle in Inner Mongolia; various *Cryptosporidium* mixed infections have been reported in cattle in China and other countries [2–4, 10, 13]. Furthermore, the same mixed infections of *Cryptosporidium* species have been detected in humans [72]. In addition to the mixed infection types observed in the current study, *C. bovis* + *C. ryanae* appeared in Guangdong [52], Shanghai [25], Xinjiang [22, 38], and Henan [61]; *C. bovis* + *C. parvum* in Shanghai [25], and Xinjiang [38, 48]; and *C. parvum + C. ryanae* in Xinjiang [48] and Henan [61]. Further, this study identified *C. bovis* + *C. ryanae* + *C. parvum* and *C. parvum* + *C. ryanae* + *C. andersoni* mixed infections. In addition, *C. bovis* + *C. andersoni* and *C. bovis* + *C. ryanae* + *C. andersoni* mixed infection was reported in Guangdong [52], whereas *C. ryanae + C. andersoni* infection appeared in Henan [61].

In the current study, cattle in Inner Mongolia were mostly found to be infected with *C. bovis* (44.37%), followed by *C. andersoni* (35.10%), *C. ryanae* (21.85%), and *C. parvum* (11.92%). Although *C. parvum* (39.4%) and *C. andersoni* (18. 8%) are the most common *Cryptosporidium* species causing infection in livestock worldwide, *C. parvums* is the most common species causing infection in cattle (54.1%) [4]. Its prevalence is different from that of the common species in China (*C. andersoni > C. bovis > C. parvum* > *C. ryanae*), as reported in the literature [2]. In this study, preweaned calves were frequently infected with *C*. *bovis*, followed by *C. parvum*, which is consistent with infection prevalence reported in the literature in China. In addition, *C. parvum* was primarily detected in preweaned calves in China [2, 5, 9, 13, 16, 53, 57, 59, 61], unlike the finding in industrialized countries where *C. parvum* occurs almost exclusively [2, 9, 13, 16, 19]. In this study, postweaned calves were mostly infected with *C. bovis*, followed by *C. ryanae*, which is consistent with the global infections reported in the literature [13, 19]; however, this finding differs from that reported by studies in China revealing that *C. andersoni* is the most abundant species and *C. bovis*, *C. ryanae*, and *C. ryanae* rarely infect cattle [2]. In the present study, *C. andersoni* was the most common cause of infection in young cattle, followed by *C. bovis*; this finding was consistent with the results reported in China and abroad [2, 13, 51]. Adult cattle were often infected with *C. andersoni*, followed by *C. bovis*, and were not infected with *C. parvum*, which is consistent with the results reported in China; moreover, no mixed infections were reported [2, 51, 55]. In this study, *C. bovis* and *C. andersoni* were the most common solitary infections, which differs from the results (*C. par*vum and *C. andersoni*) reported in the literature [4]. This is attributable to the prevalence of *C. parvum* infection in several CAFOs as well as in European and North American countries.

All *C. parvum* species obtained in the present study belonged to the subtype IIdA19G1, unlike the finding in industrialized countries where cattle were mainly infected with the IIa subtype [9, 16, 32, 33]. The IId subtype mainly occurs in lambs and goat kids in European and Middle Eastern countries [12, 16] and dairy calves in Sweden and Middle Eastern countries [73, 74]. The results of this study are consistent with other reports on *C. parvum* infections in cattle in China exclusively caused by the IId subtype. Further, IIdA19G1 is the most common subtype family in China [5, 9, 16, 20] based on the reports of studies conducted in Shanghai [25], Henan [61], Guangdong [53], Heilongjiang [49], and Hebei/Tianjin [65]. IIdA15G1, which is another common subtype family in China [5, 9, 16, 20], was reported in Ningxia [54, 55], Gansu [55], and Xinjiang [22]. In China, the subtype family IIdA14G1 was found in Xinjiang [48, 57], IIdA17G1 in Beijing [69], IIdA20G1 in Xinjiang [48] and Heilongjiang [63], and IIdA15G2 in Gansu [60].

Compared with *C. hominis*, zoonotic *C*. *parvum* causes more infections in humans [75–80]; calves are considered to be the most important contributor to zoonotic cryptosporidiosis [5]. The prevalence of *C*. *parvum* infection in dairy cattle in China has dramatically increased in recent years with an increase in their populations [16]. The *C. parvum* IIa and IId subtypes are zoonotically transmitted [1, 5, 9, 12, 31], and IIa and IId subtypes have been detected in Chinese patients [16]. Although the IIa subtype has not yet been detected in cattle in China, it has been observed in various grazing animals in several provinces, including Inner Mongolia, and is prevalent in neighboring countries of China [16]. With the development of animal husbandry, the prevalence of cryptosporidiosis in China may follow the footsteps of that in industrialized countries and become a rampant zoonotic disease in China. In addition, human infections with *C. andersoni* and *C. bovis* have been reported [1, 9]. In summary, the results of this study suggest that there is a risk of *Cryptosporidium* infection in humans caused via dairy cattle in Inner Mongolia; and biosecurity measures are urgently required to delay the spread of local *C. parvum* IId subtype and imported *C. parvum* IIa subtype and other Cryptosporidium species.

## Conclusions

To the best of our knowledge, this is the first study to report that dairy cattle in Inner Mongolia were infected with four species of *Cryptosporidium* and had mixed infections involving of two or three species. *Cryptosporidium bovis* and *C. andersoni* were identified to be dominant species infecting dairy cattle in Inner Mongolia. Further, the subtype of *C. parvum* in dairy cows was confirmed to be IIdA19G1, thereby providing a detailed information on the molecular epidemiological investigation of bovine cryptosporidiosis in this region. Further, studies on cryptosporidiosis in other animals in several regions are warranted to help in identifying and elucidating the zoonotic potential and distribution patterns of *Cryptosporidium*.

## Data Availability

All the sequences obtained in our laboratory have been uploaded to the GenBank database under the accession numbers OQ029566 to OQ029580, OP861556 to OP861567, OP861717 to OP861773, OP861775 to OP861799, OP861801 to OP861850. Reference sequence accession numbers: MH049734, KT235713, FJ897787, KC885904, JN867335, AF402285, KU852718, AB237137, KU852719, AF164491, AY873781, AY262034, DQ192501, AY873780, AM937006, KU670813, AY738188, AY873782, AY700401, AY382675, and KU852720.

## Abbreviations

gp60: 60 kDa glycoprotein
SSU rRNA: small subunit ribosomal RNA
RFLP: restriction fragmentlength polymorphism
PCR: polymerase chain reaction
CI: confidence interval
OR: odds ratio
